# Erythema Ab Igne and Its Relation to Spinal Pathology

**DOI:** 10.7759/cureus.2914

**Published:** 2018-07-03

**Authors:** MN Baig, Fergus Byrne

**Affiliations:** 1 Trauma & Orthopaedic Surgery, Galway University Hospital, Galway, IRL; 2 Trauma & Orthopaedics, Galway University Hospital, Galway, IRL

**Keywords:** erythema ab igne, tfnb

## Abstract

Erythema ab igne is a skin condition usually caused by abnormal exposure to heat in a variety of forms. While common in the past, its incidence has declined significantly to become a rare occurrence today. Today, we still see an odd case once in a while, although they have become a rarity. We describe a case of a young man who presented with erythema ab igne with a unique cause.

## Introduction

Erythema ab igne is from Latin, meaning “redness from fire,” an accurate depiction of the clinical condition [1]. Erythema ab igne is a dermatological condition characterized by localized areas of hyperpigmentation and erythema due to chronic exposure to infrared radiation, most commonly heat [2]. Its presentation can range from bruising alone to itching and burning. In the literature, it has not been identified with spinal pathology, as present in the case we discuss in this report. We describe the case of a young man who presented initially with back pain and, through home remedies, developed erythema ab igne.

## Case presentation

A 28-year-old-man presented to our outpatient department with lower back pain lasting two years and getting progressively worse. The patient stated the pain started when he tried to pick up heavy luggage. He began taking over-the-counter pain medication supplemented with applying a hot water bottle to the painful region, which provided symptomatic relief. He reported using the hot water bottle for the past few months, and he used to sleep with the hot water bottle underneath his body.

**Figure 1:**
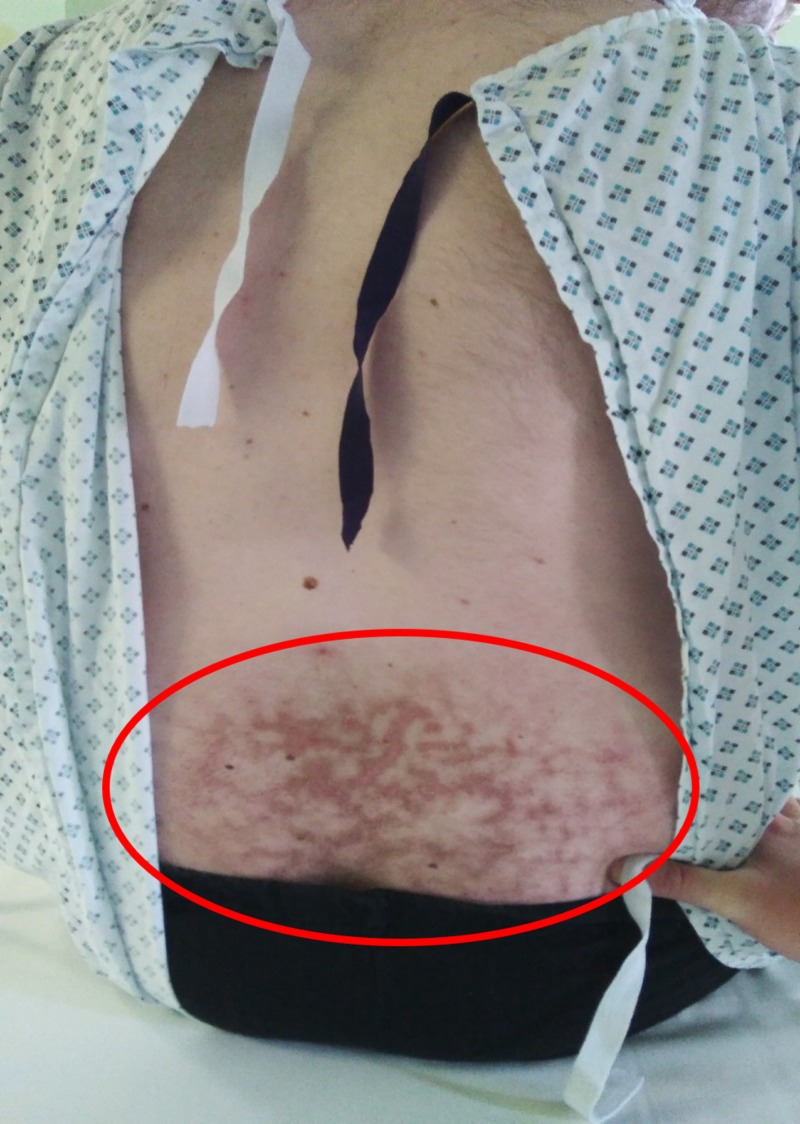
Clinical example of erythema ab igne in the lower back

The area of erythema ab igne developed a mild itch, and over next few months, the area developed hyperpigmentation (Figure 1).

**Figure 2:**
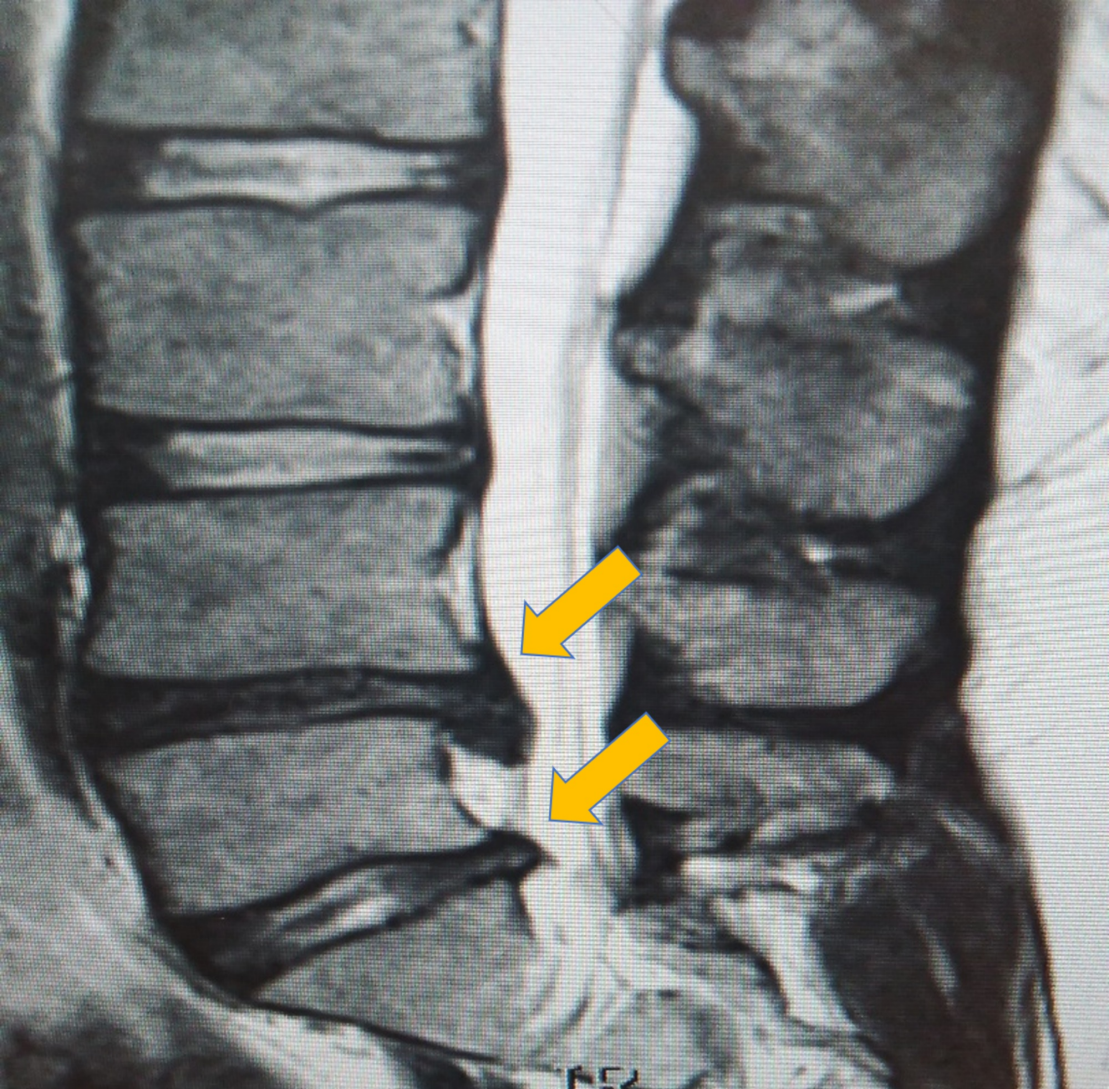
Magnetic resonance image of disc prolapse (sagittal view) Large L4-L5, L5-S1 disc bulges

He presented to our clinic with back pain. We investigated using x-rays and magnetic resonance imaging (MRI), which revealed L4-L5 and L5-S1 disc bulges (Figure 2). He was treated with a transforaminal nerve block (TFNB) injection which provided him with reasonable relief. He was told, however, that the lumbar spine pathology may require surgery later in his life.

He was advised to stop using the hot water bottle, and he was referred to a dermatologist for further evaluation and treatment.

## Discussion

Erythema ab igne is a reticulate hypermelanosis with erythema. The temperature range of the heat exposure that can cause erythema ab igne is 43°C to 47°C [3]. Historically, erythema ab igne was found in people who sit or stand close to a fire or sources of heat. Before central heating and means of modern cooking, open fires were common sources of heat for comfort and cooking food [4]. Classic causes of erythema ab igne have largely diminished in modern times. However, new, modern causes of the condition exist in the form of laptops, hot water bottles, electric blankets, and electric heaters [5].

Our case report is unique in the sense that backache is a common problem and a lot of people use the hot water bottle as a modality that gives them instant relief, is cheap, and easily available. In our case, we identify how a spinal pathology which is causing the symptom of a backache can lead to a rare dermatologic condition. Lumbar spine disc bulge is a common clinical condition which can cause severe lower back pain [6]. The treatment algorithm that is usually followed for disc bulge/prolapse is to start with oral or topical analgesia, along with physiotherapy; if that does not help, it is followed by a transforaminal nerve block injection (in nerve root compression cases). If the above-mentioned treatment options fail, then the next step is a spinal decompression. Spinal decompression can be achieved by a laminectomy or discectomy. In a lot of cases, the patients find the topical application of heat pads or hot water bottles very useful as in the above-mentioned case.

## Conclusions

Erythema ab igne is a rare condition in modern times. The recommended treatment is the removal of the source of heat. If the condition is advanced, a topical application of fluorouracil or hydroquinone can be administered as treatment. If medical treatment is refractory, photothermolysis using a neodymium-doped yttrium aluminum garnet, ruby, or alexandrite laser is used.
